# *In vivo* genome editing thrives with diversified CRISPR technologies

**DOI:** 10.24272/j.issn.2095-8137.2017.012

**Published:** 2018-03-07

**Authors:** Xun Ma, Avery Sum-Yu Wong, Hei-Yin Tam, Samuel Yung-Kin Tsui, Dittman Lai-Shun Chung, Bo Feng

**Affiliations:** 1 Key Laboratory for Regenerative Medicine in Ministry of Education, School of Biomedical Sciences, Faculty of Medicine, The Chinese University of Hong Kong, Hong Kong SAR, China; 2 Guangzhou Institute of Biomedicine and Health, Chinese Academy of Sciences, Guangzhou Guangdong 510530, China; 3 SBS Core Laboratory, CUHK Shenzhen Research Institute, Shenzhen Guangdong 518057, China

**Keywords:** CRISPR/Cas9, Genome editing, Animal models

## Abstract

Prokaryotic type II adaptive immune systems have been developed into the versatile CRISPR technology, which has been widely applied in site-specific genome editing and has revolutionized biomedical research due to its superior efficiency and flexibility. Recent studies have greatly diversified CRISPR technologies by coupling it with various DNA repair mechanisms and targeting strategies. These new advances have significantly expanded the generation of genetically modified animal models, either by including species in which targeted genetic modification could not be achieved previously, or through introducing complex genetic modifications that take multiple steps and cost years to achieve using traditional methods. Herein, we review the recent developments and applications of CRISPR-based technology in generating various animal models, and discuss the everlasting impact of this new progress on biomedical research.

## INTRODUCTION

Genome editing by manipulating functional DNA sequences in the host genome is a fundamental strategy for biomedical research. Starting from the discovery of the basic principles of DNA structure and genome organization, scientists have investigated various strategies for many decades to improve genome editing technology for different research and application purposes. 

In the 1980s, gene targeting methods emerged together with a deepening understanding of DNA repair mechanisms. Back then, DNA conversion was found to occur between homology sequences, often termed homologous recombination (HR) (Zinn & Butow, 1985). Early studies took advantage of this finding to replace a selected endogenous genome DNA segment with a foreign DNA donor carrying homology sequences in living cells (Vasquez et al., 2001). Subsequently, by combining this with mouse embryonic stem cell (ESC) technology established at the same time, traditional gene targeting technology was developed to generate genetically modified mice (Koller et al., 1989). Since 1989, genetic modification by HR-based gene targeting in living mammals has become a fundamental approach to analyze gene functions and has revolutionized our understanding of mammalian development, metabolism, and genetic diseases (Capecchi, 2005; Koller et al., 1989). 

Traditional HR-based gene targeting is associated with low efficiency and requires laborious clonal expansions and sophisticated selections to identify target cells carrying the desired modifications (Koller et al., 1989). With pioneering studies finding that the introduction of double-strand breaks (DSBs) in target DNA by rare-cutting endonuclease I-Sce-1 could increase HR efficiency by several orders of magnitude in the subsequent DNA repair process (Rouet et al., 1994), extensive effort has been made to develop programmable endonucleases.

Zinc finger nuclease (ZFN), which was first reported in 1986 as an artificial nuclease to carry a zinc finger domain and a catalytic domain from restriction enzyme FokI, was suitable for introducing DNA cleavage and enhancing HR-dependent gene targeting (Bibikova et al., 2002; Kim et al., 1996). However, the laborious work involved in the design and identification of an efficient ZFN to a newly selected target sequence significantly limited its utility. Transcription activator-like effector protein (TALE), which originated in plant pathogen *Xanthomonas sp.*, was found to recognize target DNA with highly conserved yet variable repetitive elements, each showing a preference to bind to specific nucleotides (Boch et al., 2009; Moscou & Bogdanove, 2009). Fusion of the programmable TALE domains and FokI catalytic domain thus yielded TALE-nuclease (TALEN), which is easier to construct and can introduce DNA cleavage and targeted genome modification equally efficiently as ZFN (Christian et al., 2010). 

More recently, an RNA-guided DNA-targeting approach was developed from the type II prokaryotic clustered regularly interspaced short palindromic repeats (CRISPR) adaptive immune system (Bhaya et al., 2011; Wiedenheft et al., 2012). In this system, a programmable small guide RNA (sgRNA) complexes with Cas9 nuclease and anneals with a 20-nt target DNA sequence, at the presence of the adjacent NGG PAM (proto-spacer adjacent motif) sequence in a base-pairing manner. This process allows Cas9 to introduce DSB at the target region and enables genome modification in a site-specific manner (Jinek et al., 2012). The ease of constructing a sequence-specific sgRNA and the highly specific RNA-DNA recognition has made the CRISPR/Cas9 system superior to ZFN and TALEN, becoming the most popular tool for introducing programmed DNA cleavage as well as site-specific genome modifications in cells and animals (Barrangou & Doudna, 2016; Mali et al., 2013; Ran et al., 2013). 

These recent advances in engineered nucleases, especially the CRISPR/Cas system, have opened new prospects for accomplishing robust gene targeting in previously non-permissive cell contexts. More importantly, it has widely revolutionized biomedical research by promoting quick generation of various animal models, which either carry complex genome modifications or are derived from species that could not be genetically modified previously (Dow et al., 2015; Swiech et al., 2015; Yin et al., 2016). Such progress has provided a wide range of methods as well as advanced animal models to study gene function and biological processes, significantly promoting research under *in vivo* conditions. Hence, in this review, we focus on summarizing the recent developments and applications of CRISPR-based technology in generating various animal models.

## OVERVIEW OF RECENT DEVELOPMENTS IN CRISPR-BASED ANIMAL MODELS

Since early 2013, when the first successful CRISPR-based genome editing was demonstrated in mammalian cells (Mali et al., 2013), the number of studies using the CRISPR system has grown dramatically. Among the CRISPR-based *in vivo* studies, the majority (61.2%) have been conducted using mouse models (Figure 1, left panel). With the comprehensive knowledge and technologies established so far, research investigations using CRISPR technology in mouse models have covered various areas of biomedical research, including inherited metabolic disorders (Xue et al., 2014; Yang et al., 2016), cancer (Maddalo et al., 2014; Platt et al., 2014), neurology and neuroscience (Li et al., 2015c; Swiech et al., 2015), and virus-related studies (Jiang et al., 2017; Zhu et al., 2016). 

In addition to mouse models, CRISPR-based genome editing has been demonstrated in large mammals such as pigs and monkeys to establish disease or genetic models for organ transplantation (Niu et al., 2014; Yu et al., 2016). At the same time, CRISPR/Cas9 technology has also been applied in various lower vertebrate and invertebrate models (Irion et al., 2014; Shi et al., 2015; Wen et al., 2016). The success of CRISPR technology is particularly valuable in lower vertebrate models, such as *Xenopus* and zebrafish (Irion et al., 2014; Shi et al., 2015), in which targeted genome editing could not be achieved previously. 

### Molecular mechanisms for various genome editing strategies 

Sequence-specific DNA cleavage induced by any of the above engineered nucleases will elicit endogenous cellular responses to repair the damaged DNA in target cells. Utilizing various DNA repair mechanisms to induce mutations/deletions or to incorporate insertions of foreign DNA lays the foundation for genome editing. Cellular repair of DNA damage is mediated by two main pathways, namely, homology-directed repair (HDR) and non-homologous end joining (NHEJ). Despite their varied activities in different cell types and species, both pathways are highly conserved, from yeasts to mammals (Taylor & Lehmann, 1998).

The HDR pathway mediates a strand-exchange process to repair DNA damage based on existing homologous DNA sequences (Heyer et al., 2010), allowing precise insertion of foreign DNA at target regions by replacing endogenous genomic segments with donor DNA. CRISPR/Cas9-introduced site-specific DNA cleavage triggers DNA repair and greatly promotes HR at nearby regions, thus enhancing the efficiency of HDR-based genome editing (Yang et al., 2013). In contrast, the conventional NHEJ pathway initiates DNA repair with quick occupation by the Ku70/Ku80 complex at DNA broken ends, followed by recruitment of other components for end processing and subsequently DNA ligase IV for ligation. NHEJ-based DNA repair is a homology-independent and mechanistically flexible process, which often results in random insertions or deletions (indels) of a small number of nucleotides (Lieber, 2010). Hence, CRISPR/Cas9-induced NHEJ repair has been employed to generate loss-of-function alleles in protein-coding genes (Wang et al., 2013). In general, the NHEJ pathway mediates rapid DNA repair and plays an important role in various cellular contexts. Therefore, CRISPR/Cas9-induced NHEJ repair offers high efficiency and has been exploited to develop a variety of targeting strategies. 

More recently, in addition to the conventional HDR and NHEJ pathways, studies have discovered the microhomology-mediated end joining (MMEJ) pathway, which is also termed as alternative NHEJ (Alt-NHEJ) pathway (Lieber, 2010; McVey & Lee, 2008). This MMEJ pathway repairs DNA damage by initiating single-strand resection similar to the HDR process, followed by microhomology-based alignment and ligation by DNA ligase III. In general, the MMEJ pathway mediates an error-prone repair process and plays a minor role to complement DNA repair by HDR and NHEJ (McVey & Lee, 2008). Collectively, the coupling of different DNA repair mechanisms with various strategies to design donor templates or select target sites in genomes, has resulted in a variety of targeting approaches, each having distinct advantages in different species (Table 1).

**Table 1:** Summary of CRISPR-based *in vivo* genome editing in zygotes via different DNA repair mechanisms in different species

Genome modifications and targeting strategies	Species	ESCs involvement	Efficiency*^#^	References
NHEJ-based knockout by introducing indels	Mouse: Multiple genes Single gene	No	50%–80% 8%–90%	Dow et al., 2015; Mandasari et al., 2016; Swiech et al., 2015; Wang et al., 2013; Xu et al., 2017 Challa et al., 2016; Hay et al., 2017; Helsley et al., 2016; Hinze et al., 2017; Ishikawa-Fujiwara et al., 2017; Jiang et al., 2017; Kasparek et al., 2016; Kim et al., 2017b; Li et al., 2015d; Mandasari et al., 2016; Meyer et al., 2016; Mianné et al., 2017; Miyata et al., 2016; Sweeney et al., 2017; Wang et al., 2015a; Zhang et al., 2017b; Zhong et al., 2015; Zhu et al., 2016
	Rat: Single gene	No	28.6%–45.5%	Rannals et al., 2016; Wang et al., 2016c; Yoshimi et al., 2014
	Pig: Multiple genes Single gene	No	N/A 60%–83%% for embryos 50%–100% for offspring	Wang et al., 2016a Park et al., 2017; Petersen et al., 2016; Wang et al., 2015e; Yu et al., 2016
	Monkey: Single gene	No	10.2% for offspring	Niu et al., 2014
	Sheep: Single gene	No	37.4%–87.6% for embryos 59.1%–83.3% for offspring	Crispo et al., 2015; Li et al., 2017b; Niu et al., 2017a; Zhang et al., 2017a
	Goat: Single gene	No	15%–28.6%	Malpotra et al., 2017; Wang et al., 2015c; Zhou et al., 2017
	Zebrafish: Single gene	No	~32.9%	Ablain et al., 2015; Anelli et al., 2017; Fujii et al., 2016; Gallardo et al., 2015; Gui et al., 2017; He et al., 2015; Homma et al., 2017; Hoodless et al., 2016; Lee et al., 2016; Narayanan et al., 2016; Perles et al., 2015; Shah et al., 2015; Varshney et al., 2015; Vejnar et al., 2016; Wang et al., 2014; Yuan et al., 2016a; Zhang et al., 2014
	*Drosophila:* Multiple genes Single gene	No	N/A	Port et al., 2014 Bassett et al., 2014; Gao et al., 2015; Wakabayashi et al., 2016
	Rabbit: Single gene	No	98.7% for embryos 100% for offspring	Lv et al., 2016; Yuan et al., 2016b
	Mosquito:	No	N/A	Dong et al., 2015
NHEJ-based knockout via deletion	Mouse	Yes	10%–90%	Han et al., 2014; Kraft et al., 2015; Seruggia et al., 2015; Wang et al., 2015a, 2017; Zhang et al., 2016
NHEJ-based knock-in	Zebrafish	No	4%–54%	Auer et al., 2014; Hisano et al., 2015; Kimura et al., 2014; Li et al., 2015a
	Frog	No	8%–12%	Shi et al., 2015
	Sheep	No	34.7%	Ma et al., 2017
HDR-based knockout	*Drosophila*	No	47%	Wen et al., 2016
HDR-based knock-in	Mouse: dsDNA ssODN	No	10%–88% 6%–66%	Aida et al., 2015; Chu et al., 2016; Guo et al., 2016; Han et al., 2015; Ishizu et al., 2016; Lewis et al., 2016; Li et al., 2016; Mashiko et al., 2014; Wang et al., 2015b; Wu et al., 2013 Inui et al., 2014; Zhu et al., 2017
HDR-based knock-in	Rat: ssODN	No	7.7%	Yoshimi et al., 2014
	Pig: dsDNA ssODN	No	5%–18.2% 28.6%–80%	Peng et al., 2015 Zhou et al., 2016
	Goat: ssODN	No	24%	Niu et al., 2017b
	Zebrafish dsDNA	No	1.7%–3.5%	Irion et al., 2014
	*C.elegans:* ssODN	No	4.9%–62.8%	Paix et al., 2016
	*Drosophila*	No	4.3%–10.8%	Li et al., 2015e; Lin & Potter, 2016; Liu et al., 2016; Ukken et al., 2016; Voutev & Mann, 2017; Yu et al., 2014
	Frog	No	N/A	Sakuma et al., 2016
MMEJ-based knock-in	Zebrafish	No	N/A	He et al., 2015
	Mouse	No	12%	Aida et al., 2016

*: Data were converted into percentages, without normalization or additional statistical analysis.

^#^: Data presented were based on records at the offspring stage, if stage not indicated.

### Enhanced genome editing via CRISPR-induced HDR 

HDR is a major DNA repair mechanism broadly employed in CRISPR-based genome editing (Heyer et al., 2010). In the presence of Cas9 nuclease and specific sgRNA targeting a selected sequence in the genome, site-specific DNA cleavage is introduced at the target genomic locus, which then will trigger DNA repair. When the target cells are given a large quantity of donor templates carrying homology sequences, HDR-based repair will utilize the donors as templates to repair the damaged genome, thus introducing foreign DNA included in the donor construct into the recipient genome (Heyer et al., 2010). 

The traditional gene targeting approach succeeded before the establishment of engineered nucleases. To accomplish sequence replacement in the genome, this approach relies on the HDR repair process triggered by spontaneous DNA damage that randomly occurs near target regions, (Koller et al., 1989). The desired targeting events occur at low frequency. Hence, successful genome targeting requires long homology arms in donor constructs, and needs sophisticated selection and clonal expansion in mouse ESCs before generating chimeric animals and genetically modified offspring (Koller et al., 1989; Thomas & Capecchi, 1987). It often takes more than one year to establish a knock-in or knockout strain of mouse.

Site-specific DNA breaks trigger DNA repair around a target region. Hence, coupling this to the CRISPR system can greatly enhance the efficiency of HDR-based genome targeting and result in a high success rate of desired targeting. This improvement has bypassed the usage of ESC cells, allowing direct genome targeting in mouse zygotes (Yang et al., 2013). The direct genome targeting in zygotes via CRISPR-coupled HDR can produce a high percentage of chimeric animals and genetically modified mouse strains within 3–6 months, a much shortened period of time (Yang et al., 2013). Moreover, direct genome targeting in zygotes has also overcome the limitations of ESC unavailability, and made genome editing possible in many previously inaccessible organisms, such as pigs and monkeys (Peng et al., 2015). Furthermore, the introduction of site-specific DNA breaks allows the use of much shorter homology arms to achieve successful genetic modifications. Around 1 000 bp homology fragments are usually sufficient, and around 100 bp single-stranded oligodeoxynucleotides (ssODN) carrying a 50–60 nt homology sequence at each side are effective in introducing small mutations/insertions to produce genetically modified animals (Inui et al., 2014; Zhou et al., 2016).

CRISPR-coupled HDR-mediated *in vivo* genome editing has been broadly used to introduce knock-in or knockout in the genome of various animal models for studying gene functions, modeling diseases, or developing novel treatment by correcting disease-associated mutations. Direct injection of Cas9 mRNA, sgRNA targeting only the mutant allele, and donor ssODN carrying a wild-type allele sequence into mouse zygotes carrying a heterozygous dominant-negative cataract-causing mutation in the *Crygc* gene resulted in cataract-free progeny (Wu et al., 2013). Besides rodents, large animals like pigs have also been used for disease modeling (Peng et al., 2015; Wang et al., 2015d; Zhou et al., 2016). In these studies, together with the use of the single blastocyst genotyping system and/or ssODN donors, researchers can assess sgRNA efficiency at the embryonic stage and achieve up to 80% targeting efficiency in producing animals carrying the desired genetic modification. Furthermore, successful targeting has also been reported in lower vertebrates and invertebrates (Irion et al., 2014; Li et al., 2015e; Lin & Potter, 2016; Liu et al., 2016; Paix et al., 2016; Sakuma et al., 2016; Ukken et al., 2016; Voutev & Mann, 2017; Yu et al., 2014). Targeted gene modification and tagging has been achieved in *Drosophila* based on the CRISPR/Cas9-coupled HDR approach (Li et al., 2015e; Lin & Potter, 2016; Liu et al., 2016; Ukken et al., 2016; Voutev & Mann, 2017; Yu et al., 2014), with a similar method also applied in zebrafish, producing up to 50% targeted mutations in larvae (Irion et al., 2014). With modified ssODN templates and CRISPR components, gene editing efficiency has reached 85% in *C. elegans* (Paix et al., 2016). Targeted genes or long noncoding RNA (lncRNA) can be precisely replaced with fluorescence reporters to deplete target genes by inserting visible markers (Platt et al., 2014; Wen et al., 2016).

Gene correction in somatic tissues has also been performed using the CRISPR system and donor DNA (Table 2). Targeting of deficient ornithine transcarbamylase in the mouse model showed more than 10% correction of the deficient gene in liver cells and significantly improved the survival rate in target groups (Yang et al., 2016). Similarly, somatic correction of Duchenne muscular dystrophy (DMD) caused by a mutation in the gene encoding dystrophin has been reported, showing a 70% increase in functional dystrophin and apparent improvement in the mouse model (Bengtsson et al., 2017).

**Table 2:** Summary of CRISPR-based *in vivo* genome editing in somatic tissues

Genome modifications and targeting strategies	Species	Delivery system	Efficiency*^#^	References
NHEJ-based knockout via indel formation	Mouse	Virus Hydrodynamic injection Electroporation Cell injection	14.8%–86% N/A N/A 90%	Cheng et al., 2014; Chiou et al., 2015; de Solis et al., 2016; Ding et al., 2014; El Fatimy et al., 2017; Guo et al., 2017; Heckl et al., 2014; Hung et al., 2016; Kaminski et al., 2016; Kim et al., 2017a; Li et al., 2017a; Monteys et al., 2017; Ortinski et al., 2017; Tabebordbar et al., 2016; Wang et al., 2015a, 2016b; Yin et al., 2017 Liang et al., 2017; Weber et al., 2015; Xue et al., 2014 Kalebic et al., 2016; Latella et al., 2016; Maresch et al., 2016; Shinmyo et al., 2016; Straub et al., 2014 Courtney et al., 2016; Katigbak et al., 2016; Wu et al., 2017
	Chicken	Electroporation	N/A	Véron et al., 2015
NHEJ-based knockout via deletion	Mouse	Virus Hydrodynamic injection	N/A 30%	Long et al., 2016; Nelson et al., 2016 Pankowicz et al., 2016
HDR-based knockout	Mouse	Virus	85%	Platt et al., 2014
HDR-based knock-in	Mouse	Virus Electroporation Cell injection	2.3%–6%	Bengtsson et al., 2017; Xie et al., 2016; Yang et al., 2016; Yin et al., 2016 Chen et al., 2016 Ou et al., 2016
MMEJ-based knock-in	Mouse	Virus	20%	Yao et al., 2017
NHEJ-based knock-in	Mouse	Virus	3.4%–10%	Suzuki et al., 2016
Chromosomal rearrangement	Mouse	Virus	N/A	Blasco et al., 2014; Maddalo et al., 2014

*: Data were converted into percentages, without normalization or additional statistical analysis.

^#^: Data presented were based on records at somatic tissue level, if stage not indicated.

### Diverse targeting strategies through CRISPR-induced NHEJ-mediated DNA repair

Double-strand DNA breaks due to the disruption of phosphodiester bonds between adjacent nucleotides in double-helix DNA. While HDR repairs a broad range of DNA damage, NHEJ is the primary mechanism for repairing DSBs in mammalian cells. With site-specific DSBs able to be introduced at almost any target site in the genome with high efficiency and accuracy using the CRISPR system, the NHEJ repair mechanism has been broadly employed to introduce random mutations at selected target sites. This CRISPR-coupled NHEJ-based mutagenesis approach can disrupt protein coding potential of a target gene by causing frame shift or premature termination, and therefore deplete functional proteins and introduce loss-of-function effects (Figure 1). To date, most animal models established using CRISPR technology have employed this strategy to knockout a specific gene, especially model organisms that are incompatible with the traditional HDR-based strategy, such as zebrafish or *Xenopus* (broadly noticed via personal communications) (Table 1 and Table 2) (Auer & Del Bene, 2014; Irion et al., 2014; Won & Dawid, 2017). Furthermore, due to its simple principles and procedures, CRISPR-NHEJ-based mutagenesis has been applied in high-throughput studies. Xu *et al.* reported successful loss-of-function screening to identify genes essential to tumorigenesis in mice using pre-constructed sgRNA libraries (Xu et al., 2017). Interestingly, *in vivo* application of a sgRNA library has also been reported in zebrafish (Shah et al., 2015). Combining CRISPR-based high-throughput screening with excellent accessibility to embryonic development, straight-forward phenotyping has allowed large scale analysis of gene function. Shawn M. Burgess and colleagues have verified more than 50 genes by this method (Varshney et al., 2015), and Stefania Nicolia’s team has succeeded in a similar screening using the sgRNA pool-targeting miRNA family (Narayanan et al., 2016). 

In addition, NHEJ repair has been found to be highly efficient in re-ligating DNA ends from DSBs concurrently produced by the CRISPR system at two different genome loci, despite the long distance in genome. In support of these observations, the CRISPR-coupled NHEJ repair mechanism has also been employed to delete selected large DNA fragments by targeting two regions in the same chromosome (Dow et al., 2015; Han et al., 2014; Wang et al., 2015b) or catalyzing the desired genomic rearrangements by targeting two selected regions from different chromosomes (Blasco et al., 2014). These strategies have succeeded in generating mouse models carrying a 353-kb intragenic deletion of *Laf4*, which recapitulates a human malformation syndrome (Kraft et al., 2015), and engineering mouse models that harbor chromosomal rearrangements recurrently found in lung cancer to model carcinogenesis (Blasco et al., 2014; Maddalo et al., 2014). The functional study of lncRNA genes is another important application of NHEJ-mediated large fragment deletion. Knockout of the lncRNA gene *Rian* through a large deletion of up to 23 kb demonstrated efficiency as high as 33% (Han et al., 2014) can be achieved, with similar results reported for the tyrosinase (Tyr) associated lncRNA gene (Seruggia et al., 2015).

Rather strikingly, CRISPR-coupled NHEJ repair has also enabled high-efficiency knock-in of exogenous DNA at pre-selected locations. This is consistent with common observations that NHEJ is the predominant repair mechanism in mammalian cells. Since the early 1980s, transgenic technology has been established and applied broadly to render stable ectopic expression by introducing foreign DNA fragments carrying complete gene cassettes into host genomes (Palmiter et al., 1982). Later studies have found that the NHEJ repair mechanism is responsible for capturing foreign DNA fragments at spontaneously occurring DSBs in the genome, resulting in random integrations (Lin & Waldman, 2001). Consistently, traditional gene targeting studies have also shown that the frequency of random DNA integration via the NHEJ repair mechanism is significantly higher (over 1 000-fold) than targeted insertion mediated by the HDR pathway (Vasquez et al., 2001). Due to the unavailability of programmable site-specific nucleases and their erroneous nature, the potential of the NHEJ mechanism in targeted DNA knock-in was largely neglected for a long time. 

Until recently, after ZFN was successfully established, short oligonucleotides (<100 bp) were able to be inserted efficiently at ZFN-induced DSBs via NHEJ repair (Orlando et al., 2010). Subsequently, inclusion of a ZFN or TALEN target sequence in donor vectors showed that simultaneous cleavage of donor and genome DNA could enable targeted integration via NHEJ repair (Cristea et al., 2013; Maresca et al., 2013). Using promoterless fluorescence reporters followed by direct quantification using fluorescence-activated cell sorting (FACS), we compared the frequencies of NHEJ- and HDR-mediated knock-in after coupling with the CRISPR system (He et al., 2016). We found that knock-in via CRISPR/Cas9-induced NHEJ is superior to the commonly used HDR-based method in all human cell lines examined (He et al., 2016). This NHEJ-based knock-in approach has been applied in precise reporter knock-in in zebrafish (Auer et al., 2014; Hisano et al., 2015; Irion et al., 2014; Kimura et al., 2014; Li et al., 2015a) and *Xenopus* (Shi et al., 2015), with such gene targeting previously impeded by the deficiency of the HDR pathway. More recently, CRISPR/Cas9-induced NHEJ has been shown to mediate high efficiency knock-in in mouse somatic tissues (Suzuki et al., 2016), but success in targeting zygotes or blastocysts to generate genetically modified mice has not yet been reported.

Through CRISPR-coupled NHEJ repair, various genome targeting strategies have been established and utilized in generating genetically modified animal models. From studies published since early 2013, 75.9% (110/145) of *in vivo* genome editing studies have employed NHEJ-based targeting strategies. Extensive evidence has shown that NHEJ-based genome targeting is simpler, more flexible, and more efficient compared with HDR-based approaches. Without homology sequences involved, the design and system construction for NHEJ-based strategies are less laborious. On the other hand, however, the random nature of NHEJ repair incurs disadvantages including the unpredictability of indel-based mutagenesis as well as off-target cleavage and insertion. 

### Genome editing by CRISPR-induced MMEJ repair

Distinct from NHEJ and HDR, the two common forms of DNA repair, MMEJ requires microhomologous sequences of only 5–25 bp for the repair of DSBs in DNA. Sakuma et al. devised a detailed protocol for CRISPR-based gene knock-in using MMEJ, termed Precise Integration into Target Chromosomes (PITCh) (Sakuma et al., 2016).

In this system, DSBs are needed in both the genomic DNA and donor vector to insert a DNA fragment from the donor into the genome. As MMEJ repair requires the presence of microhomology both upstream and downstream of the DSB site, two microhomologous sequences need to be added to the donor vector at both sides of the purpose sequence (Sakuma et al., 2016). For the CRISPR system, two sgRNAs are required to generate DNA cleavages near the microhomology sequences on both sides, while one sgRNA is used to induce DSBs on the genome DNA (Figure 2). Longer microhomologies of around 20 bp are currently used to improve accuracy. After alignment between microhomologous sequences, the unmatched non-homologous sequences at the 3'-parts on both sides of the donor appear as single-strand tails and are removed. This results in the loss of a small part of the genome sequence at the target sites. Therefore, MMEJ-based genome editing is associated with deletion/insertions that are often larger than NHEJ-introduced indels (Villarreal et al., 2012).

Targeted integration mediated by CRISPR-coupled MMEJ has been demonstrated in cultured cells and the generation of genetically modified zebrafish (He et al., 2015; Hisano et al., 2015; Nakade et al., 2014). Moreover, one-step knock-in of gene cassettes and floxed alleles has also been achieved in human cells and mouse zygotes through MMEJ (Aida et al., 2016). Recently, precisely targeted gene integration in somatic tissues to correct mutation of the *Fah* gene and rescue liver failure in *Fah−/−* mice has also been demonstrated (Yao et al., 2017). 

### Comparison between different targeting strategies 

Conventional NHEJ repair does not require the presence of homology sequences and involves minimal processing of DNA broken ends. The activity of the NHEJ pathway is high and stable throughout the cell cycle. Distinctly, the HDR repair mechanism relies on long homology sequences (> 500 bp in general) to repair DNA lesions, and is only active from the late S phase to G2 phase during the cell cycle. The MMEJ pathway depends on microhomology sequences (5–25 bps) for DSB repair and is active during the M to early S phase (Taleei & Nikjoo, 2013). These differences explain why the activities of the different DNA repair pathways vary in different cell contexts. 

The intrinsic activities of the two major pathways, HDR and NHEJ, also vary in different species, despite high conservation of these pathways across a broad range of organisms. Lower vertebrates, such as zebrafish and *Xenopus*, are deficient in HDR-based DNA repair. Hence, modification of genome sequences in these models has mainly succeeded with NHEJ-based strategies, such as transgenesis, indel-based targeted mutagenesis/deletion, or the recent knock-in approach based on coupling TALEN- or CRISPR-induced DNA cleavage to the NHEJ repair mechanism (Auer et al., 2014; Hisano et al., 2015; Irion et al., 2014; Kimura et al., 2014; Li et al., 2015a; Shi et al., 2015) (Table 1). In mammalian systems, although HDR was first employed to produce genetically modified mice, evidence shows that the NHEJ repair mechanism is predominant (Vasquez et al., 2001). Thus, the efficiency of NHEJ-based genome editing is generally superior to HDR-based approaches (He et al., 2016). 

Scientists have attempted to manipulate the balance between the HDR and NHEJ pathways. Through inhibiting DNA ligase IV, a key component of the NHEJ pathway, studies have shown that the efficiency of HDR-based gene targeting can be increased substantially (Chu et al., 2015). Similarly, silencing KU70, KU80, or DNA ligase IV largely suppressed NHEJ-mediated introduction of indels at the junction and enhanced HDR-mediated genome editing (Pierce et al., 2001). To date, this type of approach has not been applied for *in vivo* gene targeting.

Besides efficiency, accuracy is another major concern. The HDR-based targeting strategy requires homology sequences as a template for DNA replication to repair induced DNA cleavage. It involves the cloning of homologous DNA and multi-step construction of donor plasmids. In return, the designed modifications can be introduced into the genome with high accuracy and off-target integrations can be largely reduced compared to other knock-in strategies. MMEJ-based targeting requires microhomologous sequences, which can be easily introduced into donor vectors through synthesized oligos, or during PCR amplification of the desired DNA for insertion. Although the intrinsic MMEJ pathway often plays a minor role in overall DNA repair, the MMEJ-based targeting strategy has shown efficiency up to 10-fold higher than that of the HDR-based approach (Yao et al., 2017). Lastly, the NHEJ repair mechanism, which is completely independent of any homology sequences, offers the easiest path to modify an existing design for a new target site in the genome. In our recent study, a universal donor was established with the use of artificial sgRNA ,which did not target any sequence in mice and humans (He et al., 2016). With the minimum work involved in constructing the new sgRNA to the genome, the whole system was easily orientated for targeting a new locus (He et al., 2016). However, the random errors potentially present at the integration/ repair junctions with NHEJ-based targeting approaches should be considered during the design. 

## CONCLUSIONS

The recent advent of CRISPR technology has offered the simplest and possibly ultimate solution for introducing site-specific DSBs in genome DNA, which was once an insurmountable challenge in genome editing. Through coupling with different DNA repair mechanisms present in the endogenous cellular system, various targeting strategies have been developed to introduce a wide range of modifications in the genome through sequence-based editing. While further research is needed to evaluate the off-target issues and overcome the risks by developing improved CRISPR systems, the above technological advances have undoubtedly revolutionized biomedical research. The CRISPR-based genome editing approaches have significantly promoted studies on gene function via the rapid generation of animal models that carry genetic deficiencies of single or multiple genes. In addition, they have also enabled modeling of genetic diseases caused by chromosomal rearrangement or large deletions. Therefore, rapid progress could be foreseen in establishing various animal models for disease modeling or therapeutic intervention, which will significantly improve our understanding of human diseases and promote the development of new therapeutic strategies. 
